# Nail unit mystery: Atypical hemangioma presentation

**DOI:** 10.1016/j.jdcr.2025.02.026

**Published:** 2025-03-11

**Authors:** Andrea B. Lastra-Annexy, Eduardo Michelen-Gómez, Julian O. Barrera-Llaurador, Julio Sánchez, Alma M. Cruz-Santana

**Affiliations:** a Universidad Central del Caribe, Bayamón, Puerto Rico; b Department of Public Health, University of Puerto Rico, Medical Sciences Campus, San Juan, Puerto Rico; c Department of Dermatology, University of Puerto Rico, Medical Sciences Campus, San Juan, Puerto Rico

**Keywords:** hemangioma, pathology, vascular proliferation

## Introduction

Vascular proliferations encompass a diverse group of benign and malignant lesions characterized by the abnormal growth of blood vessels. Among these, hemangiomas represent the most common vascular tumors, particularly prevalent in pediatric populations and relatively rare in adults.^1^ In adults, hemangiomas most commonly appear on the skin, particularly on the face, neck, and trunk. Pathologically, these lesions are characterized by an abnormal proliferation of blood vessels, which can vary from well-circumscribed, capillary dense areas to cavernous structures filled with blood. Clinically, they present as red to purple nodules or plaques, which may be asymptomatic or cause cosmetic concerns and, occasionally, discomfort or ulceration. Despite their benign nature, adult hemangiomas warrant careful evaluation to differentiate them from other vascular anomalies and potential malignancies. Hemangiomas are classified into several subtypes, including capillary hemangioma, cavernous hemangioma, and atypical variants such as hobnail hemangioma. Hobnail hemangioma, a relatively rare subtype, is notable for its distinctive histologic features, including hobnail endothelial cells and a prominent vascular component, making its recognition crucial for accurate diagnosis and management.^2^ This case report aims to highlight a patient with a hemangioma and the possibility of its subtype, hobnail hemangioma, emphasizing the importance of histopathologic evaluation.

## Case

A 62-year-old man with no significant past medical history and no known drug allergies presents to the Dermatology Clinic at the Medical Sciences Campus of the University of Puerto Rico for evaluation of a painful mass on his fingernail. This mass first became apparent after his nail fell off due to trauma, from a heavy object falling on his hand at work (Table I, row 2). Before the incident, the patient noted that the affected nail had always been distinctive; it was thicker and yellow compared with his other nails and had a curved growth pattern (Table I, row 1).

**Table I:** Physical examination

Clinical description and timeline	Image
(1) Thickened nail with a yellowish-brown hue and slight curvature on the second fingernail of left hand	
(2) Soon after, the nail fell off, revealing an erythematous nodule that extends from the nail matrix to the nail bed on the second fingernail of left hand	
(3) Initial visit—Erythematous, ulcerated, friable nodule that extends from the nail matrix to the nail bed on the second fingernail of left hand	
(4) Follow-up visit—Status after shave biopsy and regrowing nail tissue	

On physical examination, the patient exhibited an erythematous, friable nodule extending from the nail matrix to the nail bed of the second fingernail on his left hand (Table I, row 3). He expressed concern about occasional spontaneous bleeding episodes accompanied by tenderness. During the initial visit, a shave biopsy was performed without complications, and local wound care was recommended. The biopsy revealed a tumor composed of irregular blood vessels lined by flat endothelial cells in the dermis (Table II, row 1). Deeper sections showed an ulcer, and immunohistochemical analysis revealed no evidence of melanocytic or keratinocytic proliferation (Table II, row 2, image a). A diagnosis of hemangioma of the skin and subcutaneous tissue was confirmed, and reassurance along with observation was recommended at the follow-up visit, during which we observed regrowing nail tissue status after shave biopsy (Table I, row 4).

**Table II:** Pathology images

Stains	Pathology
(1) Hematoxylin-eosin	a.
(2) Deeper section	a. b. c.
(3) D2-40	a. b. c.

Immunohistochemical tests of this lesion reveals dilated vascular channels positive for CD31, CD34, and smooth muscle actin, with a few vessels also positive for D2-40, and negative for GLUT-1, Factor XIIIa, Cytokeratin 7, Melan-A, S100, KI-67, SOX-10, and Cytokeratin AE1/A. The presence of CD31 and CD34 indicate a endothelial cell origin, consistent with hemangioma, whereas positive smooth muscle actin suggest smooth muscle involvement. In this particular specimen, the vascular architecture exhibited larger, more prominent blood vessels that are not clustered nor arranged in a characteristic lobular configuration, which would be typically seen in pyogenic granulomas. In addition, D2-40 is positive (Table II, row 3, images a,b, and c), whereas pyogenic granulomas do not stain positive for D2-40, thus ruling out a diagnosis of pyogenic granuloma.^3^ Another differential diagnosis to keep in mind for positive CD31 and CD34 stains would be a hobnail hemangioma, whose key histologic feature is the presence of hobnail-shaped endothelial cells lining the vascular spaces, as evidenced in Table II, deeper section images b and c. These cells have a characteristic appearance with a prominent cytoplasm and a distinct nucleus.^4^

The negative GLUT-1 effectively rules out infantile hemangioma, and negative Factor XIIIa helps exclude more aggressive vascular tumors such as angiosarcoma. The limited positivity for D2-40 may hint at lymphatic differentiation and can hint toward angiolymphoid hyperplasia with eosinophilia. However, no eosinophils were observed or no lymphoid hyperplasia, excluding that particular differential diagnosis. Additionally, deeper sections were ordered, revealing an ulcer but no evidence of melanocytic or keratinocytic proliferation, which rules out squamous cell carcinoma and melanoma.^3^ Overall, these findings collectively support a vascular proliferation with a lymphatic differentiation most likely an acquired vascular malformation secondary to trauma, similar to that of a hobnail hemangioma, which is also associated to trauma.^4^

## Discussion

Clinically, in the differential diagnosis of a lesion on the skin and subcutaneous tissue of a nail, several conditions must be considered. These include squamous cell carcinoma, amelanotic melanoma, pyogenic granuloma, and any subtype of hemangioma. Squamous cell carcinoma presents as a persistent, scaly, or ulcerated nodule and pathologically, it shows atypical keratinocytes with potential invasion into the dermis.^5^ Amelanotic melanoma, although rare, is a significant consideration due to its aggressive nature; it appears as a nonpigmented, irregular lesion, with histopathology revealing atypical melanocytes in a nested or single-cell pattern within the epidermis and dermis.^6^ Pyogenic granulomas are benign, rapidly growing lesions that appear as erythematous, friable nodules, often after minor trauma, with histopathology showing lobular capillary proliferation with inflammation and edema.^7^ In contrast, hemangiomas of the skin and subcutaneous tissue typically present as soft, erythematous to violaceous nodules; histopathologically showing proliferation of capillary or cavernous vascular channels filled with blood.^4^ Ultimately, accurate differentiation among these conditions hinges on a comprehensive patient history and the histopathologic characteristics of the lesion.

This case aims to highlight the diagnostic complexity of vascular proliferations, specifically hemangiomas, in adults. The patient’s presentation, together with histopathologic and immunohistochemical findings, suggest a diagnosis of hemangioma, with particular consideration of the rare hobnail hemangioma subtype. The distinct histologic features, including hobnail-shaped endothelial cells, support this diagnosis and help differentiate it from other vascular and nonvascular lesions. This case underscores the importance of thorough clinical history taking and pathologic assessment to guide accurate diagnosis and optimal adequate treatment.

## Conflicts of interest

None disclosed.
